# The Dutch see Red: (in)formal science advisory bodies during the COVID-19 pandemic

**DOI:** 10.1057/s41599-022-01478-w

**Published:** 2022-12-24

**Authors:** Janne Aarts, Eva Gerth, David Ludwig, Harro Maat, Phil Macnaghten

**Affiliations:** Knowledge, Technology and Innovation Group, Wageningen University & Research, Hollandseweg 1, 6706 KN Wageningen, the Netherlands

**Keywords:** Science, technology and society, Social policy

## Abstract

We analyse the roles, dynamics and logic of science advice in structuring the Dutch response to the COVID-19 pandemic, from January 2020 to December 2020. We address how the Dutch government responded by paying attention to styles of governance and expert advice. We argue that the Dutch response was shaped by the interplay of corporatist, deliberative and neoliberal forms of governance, in particular, how early corporatist tendencies seemed to create consensus during the first phase of the pandemic but quickly led to criticism and tension, most visibly at the onset of the second wave, as corporatist and neoliberal responses conflicted with deliberative and pluralist political engagement. Situating different science advisory bodies in this dynamic, we highlight how science–policy interactions and conflicts that evolved with the dynamics of the pandemic can be understood within this triad and as reflective broadly of the endurance of the Dutch model of polder governance.

## Introduction

As Jasanoff (2005) argues, science-based controversies rarely follow a purely rationalist model of science-based policy informed by rigorously established objective evidence. Rather, it is the political culture of a nation in combination with existing organisational formats for science–policy interaction that are key to understanding institutional responses to controversial science and technology – and thus why issues of concern can become issues of overt controversy and contestation. In our research, we analyse the roles, dynamics and logic of science advice in structuring the Dutch response to the COVID-19 pandemic, from January 2020 to December 2020. In particular, we delineate how the political culture of the Netherlands shaped institutional responses to the pandemic. What were the characteristics of governing and science advice in the Netherlands, and in what ways did dominant imaginaries of citizen–state relations and science advice also characterise the Dutch response to the COVID-19 pandemic? It is these questions that structure our analysis below.

Section “The macro level: national styles of governing and the Dutch science–policy interface” sets the stage through a general account of science advice and governance in the Netherlands. We describe the Dutch style and structures of policymaking as they emerged at the beginning of this century, characterised by Willem Halffman and Rob Hoppe (2005) as the coexistence of corporatist, neoliberal and deliberative arrangements. We further conceptualise science advice and health governance in the Netherlands based on studies of science-policy interaction in the Dutch health sector. Section “The meso level: the Dutch playbook for responding to pandemics” addresses how this style of policymaking shaped the institutional design for responding to pandemics in the Netherlands at the national level—the official Dutch playbook. We identify the main science advisory bodies, their functions and roles in decision-making, their disciplinary composition and how each addresses the interplay between science and policy in the coordination of expert advice. Particular attention is given to the conditions under which responses to a pandemic emerge, how scientific advice is formulated and to whom, and the networks of actors that handle the interplay between scientific and policy advice.

In the section “The micro level: science advice during the COVID-19 pandemic”, we describe the science advisory processes and decisions that took place during the COVID-19 pandemic, following the chronology of events and the extent to which the playbook was followed in practice. While the detailed timeline can be found in the Annex, in this section we identify the different styles of governing that characterised the three phases by which the pandemic unfolded: a first wave of high incidence and high uncertainty in March and April 2020, a speculation and exploration phase over the summer period when incidence or case count temporarily dropped, and the second wave of late 2020. We address the role of scientific advisory bodies during these phases, focusing on the role of the Outbreak Management Team (OMT) as the formal body for providing advice on how to control the pandemic, and the formation and role of a shadow advisory body, the Red Team, as a partial response to the perceived need for the greater inclusion of diverse perspectives. In the section “Formal and shadow science advice”, we analyse the tensions and conflicts that took place between the OMT and the Red Team. We examine how the introduction of the Red Team restructured the politics of scientific advice. We illustrate these tensions with a short case study on the controversy surrounding scientific advice on the wearing of facemasks. We conclude with a short set of reflections on the various conceptual framings and how best to characterise the Dutch response to the COVID-19 pandemic in terms of science–policy interactions.

The method adopted in this paper is a literature review of secondary sources. Official government policies and procedures of the Dutch government were examined using data published on the website of the Tweede Kamer (Second Chamber of Parliament) and the Rijksoverheid (Central Government). A keyword search was conducted: [OMT], [COVID-19], [Timeframe: 01-01-2020– 01-11-2020]. This yielded 248 results which included letters to parliament, questions of parliamentarians, reports, publications, media texts, regulations, annual plans, flyers, memos and Wob-requests (Dutch Public Access to Government Information Act). The results can be found in the additional excel sheet file ‘Policy document timeline’. All OMT meetings were also examined until the 4th of January 2021. In addition, online media articles were examined from Dutch news websites. Our research is part of the cross-national ESCaPE (Evaluating Scientific Advice in a Pandemic Emergency) research project that aims to understand how expert advice has been developed and used to govern the COVID-19 pandemic from a cross-national perspective.

## The macro level: national styles of governing and the Dutch science–policy interface

The political system of the Netherlands provides the general institutional context for the way in which scientific advice is arranged. The multi-party system prevalent in the Netherlands has never resulted in a majority for a single party, leading to coalition cabinets that over the past 12 years have been led by the right-wing liberal party, the People’s Party for Freedom and Democracy (Volkspartij voor Vrijheid en Democratie, VVD). However, the political positions of coalition partners hardly ever coalesce and much of the negotiation is done behind the scenes. Out of sight from parliament and the general public, contentious issues can be depoliticised and agreements, allegedly, forged on rational grounds, where cooperation between political elites is a recognised factor in maintaining a stable system in a socially divided society (Bogaards, 2020). Known as consociationalism (Lijphart, 1969), this enduring system of governance has important implications for scientific advice.

The behind-the-scenes negotiations are often extended to other social interest groups. In particular for economic policy, trade unions and business representatives are frequently invited as legally equal pillars or blocs of social organisations. This strategy has become known as the polder model (Oudenapsen and Mellink, 2021). Often misconceived as a democratic institution, the ‘polder tradition’ is something in between a lobby machine for interest groups, an extra-parliamentary arena for national policy-making and an informal institutional structure and ‘atmosphere’ of pragmatic collaboration and deliberation aimed at a consensus in which (designated) parties are more or less equal. Clearly, getting a seat at the negotiation table implies a privileged position for getting your points across. Science advice benefits from this system in two ways. Firstly, science provides important input to the extra-parliamentary policy-making process, directly when scientists are invited to join meetings, and indirectly through an overall reliance on science-based models and arguments (Van Dooren and Noordegraaf, 2020). Secondly, and most crucially, the government formalised the polder model for key policy areas by setting up advisory bodies. Over the course of the 20th century the number of advisory bodies grew to over a hundred (Pattyn and Timmermanns, 2022). Originally representing different social and economic sectors, the core advisory bodies have become increasingly populated by scientific experts (Halffman and Hoppe, 2005; Timmermans and Scholten, 2006). By the 1990s, these were reorganised into a reduced number of research-based institutes commonly referred to as ‘planning bureaus’ and councils. Some of the institutes are involved in policy implementation, an example being the National Institute for Public Health and the Environment (Rijksinstituut voor Volksgezondheid en Milieu or RIVM) which plays a coordinating role in vaccine campaigns. All advisory bodies are funded by the government and linked to particular ministries, which ensures a close relationship between technical experts and policymakers. Even though planning bureaus are seen as a governmental resource, their advice and predictions are generally accepted as legitimate. Tensions between different planning bureaus exist as they can compete for resources and even come up with different advice on a policy issue. Playbooks, as detailed further below, play a coordinating mechanism for science–policy interaction, even if their precise formulation can include certain advisory bodies and exclude others.

The mergers and reduction of government advisory bodies in the late 1990s had important consequences for science–policy interactions. Rooted in the small state philosophy, expert advice as well as its application to policy making became increasingly outsourced to independent expert bodies and policy consultants, regulated by the market. This neoliberal pattern entails that expertise is removed from the state and that the market is used as a means to coordinate expertise (Halffman and Hoppe, 2005; Pattyn and Timmermans, 2022). Moreover, the small-state philosophy also implies that policy-making at the national level becomes less specific. In other words, formulation and implementation of policy are increasingly happening at meso and micro levels, involving a wide variety of public and private actors (Bekker et al., 2010; Wensing et al., 2012). This combination of corporatist and neoliberal arrangements can be labelled as neo-corporatist and is prevalent in several other European countries including Austria and Switzerland (Hermann et al., 2017).

Alongside the neo-corporatist constellation, a third, deliberative pattern of public expertise has developed in which science–policy interactions function as a collective resource in facilitating public debate, as part of a shift towards more participatory and deliberative policy processes in the Netherlands. This takes shape when experts join debates with politicians and other stakeholders in parliamentary hearings, sectoral forums or the media. This requires a large degree of public participation and the availability of accessible knowledge. It also engenders a reflexive attitude towards the possibilities and limitations of expert knowledge and therefore the inclusion of a plurality of knowledge. Deliberation works out differently at different levels. At the macro level, the national parliament and public media are the main deliberative fora, both operating on the basis of rather unspecific representations. Science advice figures prominently in these domains. Arguably, the mentioned polder structure is another national platform for deliberation through representation. At the meso and micro levels deliberative arrangements of public expertise are commonly organised as ‘knowledge centres’ that vary in shape from a website to a fully-fledged research institute and present themselves as facilitators of public learning targeted at practitioners (Halffman and Hoppe, 2005). Knowledge centres focus on specific domains and issues, in the health sector typically a disease category in which the ‘public’ is largely formed by the patient group.

The three arrangements of governing science advice highlighted above inform the institutional dynamics at play in the Dutch response to the COVID crisis at the macro level. Following Pattyn et al. (2021), our analysis follows a historical-institutionalist approach that seeks to identify how the macro (administrative cultures and traditions) shaped the meso (existing and newly created structures of governance) and micro (actor strategies and negotiations) contexts (and vice versa), and how their intersections co-constituted the specificities of the response of crisis management. The approach is temporal in how it covers the passage of response from January 2020 to December 2020, a period during which decisions of grave importance had to be made at speed and when facts were few (Phase 1 of the crisis), and later when scientific responses had become more diversified (following, in particular, the formation of the Red Team) and where policy-makers and politicians had more opportunity to ‘shop around’ (Phase 2 of the controversy) (see also Hodges et al., 2022).

## The meso level: the Dutch playbook for responding to pandemics

The Netherlands has a system in place for different levels of disease outbreaks. The 2008 Public Health Act sets out national collaboration structures designed to regulate disease control measures and provide an institutional framework to distribute responsibilities in the face of large-scale infectious disease outbreaks. For outbreaks that stay within regional borders, the prevention and control of infectious diseases are the joint responsibility of an internal regional network consisting of the Board of Mayors, the City Council Members, the so-called Safety Regions and the Ministry of Health, Welfare and Sports (VWS). Once an outbreak exceeds regional borders, responsibility moves mainly to the Safety Region, consisting of part of a coordination unit for medical assistance (GHOR). In response to national crises, the Ministry of Health, Welfare and Sports (VWS) holds decision-making power over which legislations and regulations get implemented at a national level. The Netherlands consists of 25 Municipal Health Services (GGD) regions that have responsibility for implementing infectious disease control measures. In the Dutch playbook for responding to pandemics, the focus is placed on the national operating systems for managing crises such as infectious disease outbreaks (RIVM, 2020a).

### The science advisory actors pre-COVID-19

The RIVM (National Institute for Public Health and the Environment) has responsibility for infectious diseases and vaccinology, environment and safety, and public health and health services (RIVM, 2020b). The RIVM combines research for policy with policy implementation through the coordination of prevention and control responses. RIVM studies are mainly commissioned by Dutch ministries and tackle issues related to its core mission of ‘work[ing] towards a healthy population living in a sustainable, safe and healthy living environment’. The RIVM steps in if there is an acknowledged problem within its domain of responsibility, if there are differing opinions about the solution to the problem, if it is a problem that must be resolved through collective action or by political decision-making, and if the issue is being covered by the media (RIVM, 2020b). A sub-unit of the RIVM is the Centre for Infectious Disease Control (CIb), with responsibilities for providing policy advice to the Dutch Government and supporting professionals in health care and public health. The main task of the CIb is to coordinate and communicate control measures in conjunction with local and regional authorities, develop policy advice on the prevention and control of infectious diseases, and advise the government and healthcare professionals on policy implementation (RIVM, 2020c). In the event of major calamities that require decisions from the government and international outbreaks of infectious diseases, the RIVM–CIb sets up an Outbreak Management Team.

The Outbreak Management Team (OMT) is an emergency organisation that is constituted when existing guidelines or playbooks do not provide sufficient grounds for robust decision-making, whose permanent members include the director of the RIVM-CIb (as Chair), the head of the RIVM National Coordination Centre for Communicable Disease Control (LCI) (as Secretary), representatives from the Dutch College of General Practitioners (NHG), the Netherlands Center for Occupational Diseases (NCvB), the Dutch Society of Medical Microbiology (NVMM), the Infectious Diseases Society of the Netherlands (VIZ), and the National Consultation on Infectious Disease Control (LOI), alongside ad hoc members who can include virologists, internists, gynaecologists, paediatricians, pulmonologists, epidemiologists and other medical specialists. The Outbreak Management Team aims to provide a space in which participants operate in a personal capacity, freely able to share expertise and arrive at the best advice for decision-making.

### The Dutch playbook on pandemics pre-COVID-19

The Dutch structure to handle pandemics, as described above, operates according to a pre-arranged procedure, the schematic of which consists of several steps.

#### Step 1

The National Institute for Public Health and the Environment (RIVM) calls for coordinated advice on outbreaks or potential threats of infectious diseases. The RIVM Centre for Infectious Disease Control (RIVM–CIb) and the RIVM analyse and evaluate potential risks to public health together with partners. At this stage their role is to advise the Minister of Health, Welfare and Sports (VWS) quickly and adequately, and for the director of the RIVM–CIb to determine whether to instigate an Outbreak Management Team (OMT). Before the OMT is constituted, the RIVM–CIb maps out the crisis and conducts a systematic evaluation (RIVM, 2020d, 2020e).

#### Step 2

The mission of the Outbreak Management Team is to give the ‘best possible professional advice’ to responsible decision-makers, who assess the advice based on the counsel of the Policy Advisory Committee (BAO) for administrative and political feasibility. The OMT provides risk estimation, based on computer-modelled scenarios, risk reduction options, and the magnitude of achievable results, including an estimation of the level of (un)certainty of the risks and of the effectiveness (and potential costs) of proposed measures. The OMT provides expertise on risk analysis and on measures to control infectious diseases. The negotiations of the OMT are confidential to help ensure that experts can share ideas freely.

#### Step 3

The Outbreak Management Team formulates recommendations which are sent to the Policy Advisory Committee (BAO). If the advice is not unanimous, the OMT lays out different aspects of multiple decision paths. In the confidential report, considerations and points of discussion which arose during the meeting are included. The Director-General of Public Health (DGV) at the Ministry of Health, Welfare and Sport (VWS) has the authority to gather a Policy Advisory Committee (BAO) to consider the advice of the OMT based on political and administrative feasibility. The involved ministers decide if the Policy Advisory Committee’s advice is to be implemented. The dissemination of information to the public at this stage is restricted.

#### Step 4

The Minister of Health, Welfare and Sport (VWS) decides if and how to implement the advice, which is operationalised by the RIVM Centre for Infectious Disease Control (RIVM–CIb) in the form of a set of guidelines for GGDs and health care professionals. Only after the Minister has issued a decision, will the advice from the OMT and the BAO be published. The RIVM–CIb coordinates the communication of decisions to professionals, doctors, and social care workers of the Municipal Health Services (GGDs) (RIVM, 2020e).

## The micro level: science advice during the COVID-19 pandemic

The Dutch playbook as codified in the 2008 Public Health Act gives guidance to the OMT in an infectious disease outbreak event. The playbook is characterised by applying a top-down approach following an (local) outbreak or threat that has been upscaled to the national level. During the first months of the COVID-19 pandemic the playbook was put into practice and rarely challenged. In March/ April 2020, during the early phase of the crisis, the playbook proved an efficient way to put national rules and measures into practice. Public areas were closed, most facilities and offices moved their activities into online spheres, etc., and Dutch life was slowed down. In response, there was widespread compliance and acceptance from Dutch citizens towards the restrictions. In the sections below we present a timeline of events (presented in more detail in the Supplementary Information File) and how the typologies of science governance can help explain these.

### The First Wave: January 2020–June 2020: The period of the Dutch corporatist tradition

The first OMT meeting to discuss the pandemic was held on 24 January 2020 and included a briefing of available information and a description of the background and the situation in Wuhan. The risk for the Netherlands was classified as ‘moderate’ in the meeting, the risk of transmission being regarded as not high since travel in and out of China had been cancelled. On 29 January 2020, the procedure for an A-level disease outbreak was initiated. On 3 March, the Ministerial Crisis Management Committee (MCCb), chaired by the prime minister and substantially taking over lead responsibility for crisis response from the VWS, adopted a set of measures aimed at preventing the further spread of the coronavirus. Prime Minister Mark Rutte gave a speech on 16 March, addressing possible strategies, including that of herd immunity, a total lockdown, or no intervention at all. He expressed his preference for the controlled spread of the virus among non-vulnerable groups until the virus had stopped spreading and a large part of the population had acquired immunity. This was framed by the government as an ‘intelligent lockdown’ and implemented on 23 March. This call relied on the practical ‘common sense’ of the Dutch public and their willingness and capacity to follow guidelines in ways that allowed basic facilities to remain open, facilitated some movement of people and that appealed to people’s own sense of morality and liberty. The reason not to impose a total lockdown was explained by OMT chair Jaap van Dissel and Prime Minister Rutte as follows: whereas a total lockdown was seen as having the effect of stopping the transmission of the virus it would also stop the Dutch population from building immunity; by contrast, with an intelligent lockdown, the young and healthy population would become infected in an orderly manner, with population immunity building up and the elderly and vulnerable groups protected. While this speech received significant backlash from societal groups and the media, as such a policy would potentially cost lives (Holdert, 2020; Holdert et al. 2020), it is equally clear that politicians may have needed a reassuring narrative to back the delay of drastic measures such as a lockdown in Spring 2020. A managed herd immunity strategy through natural infection was based on (what in retrospect could be viewed as) a naïve belief that mild COVID-19 infections would help lessen the impact of the pandemic and leave fewer deaths in its wake, at least in the longer term (Farrar, 2021). However, politicians and their advisers should not be blamed too heavily for this naivety. The uncertainty about the impacts of the pandemic in early 2020, even amongst medical experts, was so high that it is entirely understandable for politicians to try to offer a narrative with a positive tone.^1^

Rutte stressed in public that he did not want to ‘be the boss’, arguing that Dutch citizens should be responsible for their own behaviour. Rutte delegated tasks to the Dutch population on how to limit social contacts, both by making use of common sense and by instigating non-mandatory legislation with phrases such as ‘as little as possible’ and ‘we urgently advise’. Korteweg (2020) called this a ‘stretchy policy’ and saw continuity in Rutte’s devolved approach with a more general evasive strategy of the Prime Minister toward complicated and sensitive policy issues. Alternatively, such a policy can be seen as in line with a deliberative (and egalitarian) Dutch political culture that appeals to people’s own responsibility and self-discipline and their desire not to be told ‘what to do’ (Pattyn et al., 2021). Such an approach fits with a seasoned politician’s attempt to present plausible narratives and arguments that chime with the public mood. This skill of ‘sense-making’ is critical in encouraging citizens to comply with measures, and to ‘follow the rules’, that inevitably impact citizen’s liberty and ways of life (Hodges et al., 2022). As the COVID-19 pandemic continued, Rutte admitted that his decentralised approach and emphasis on individual responsibility had not always had the intended effect and that the communication had been on occasion unclear (Ast and Winterman, 2020).

**Fig. 1:**
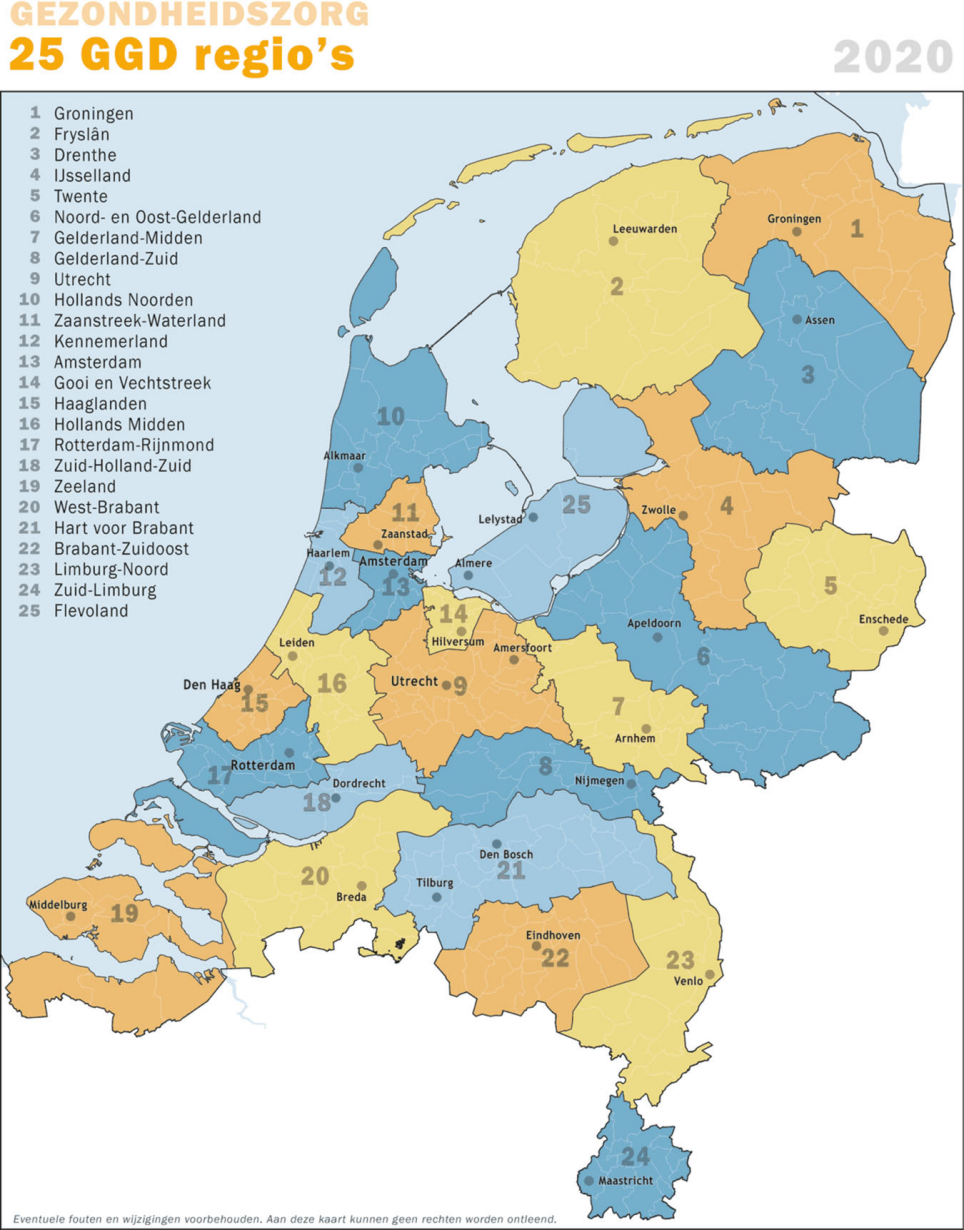
The 25 GGD Municipal Health Service regions in the Netherlands.

**Fig. 2:**
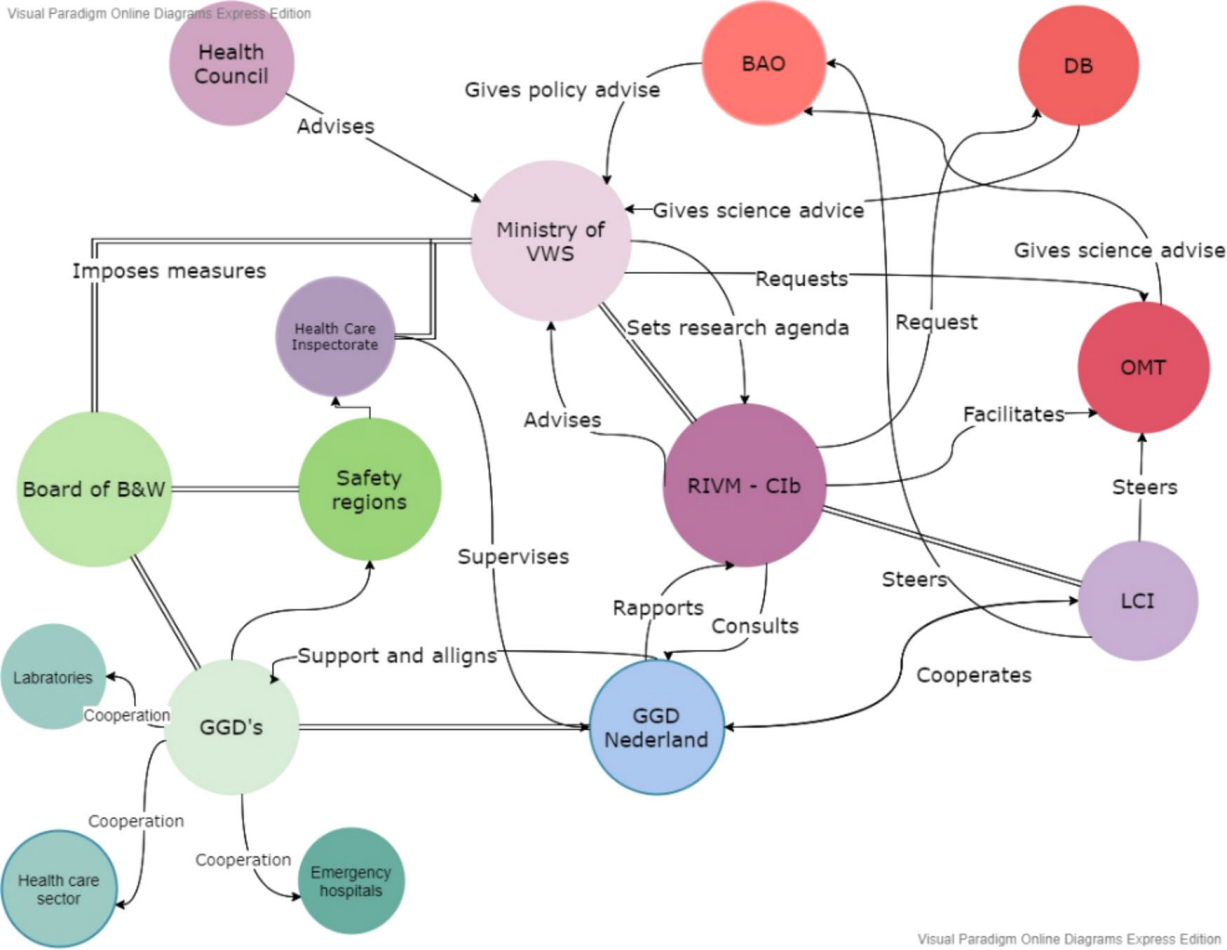
Network of actors in the Netherlands in response to a pandemic.

**Fig. 3:**
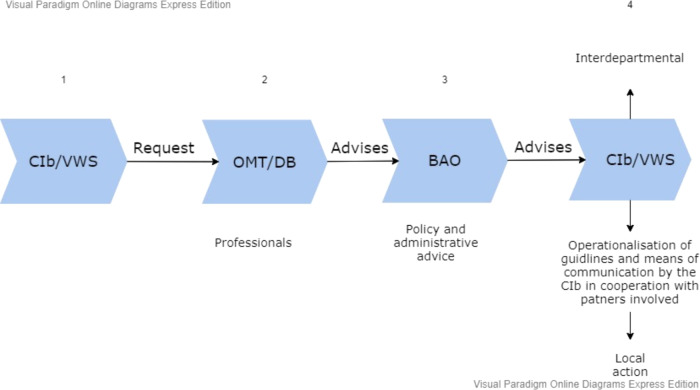
Decision-making process in the Netherlands in response to a pandemic.

At the beginning of April 2020, the OMT stressed the importance of contact tracing. Testing and protecting the elderly and vulnerable groups were cardinal priorities at this point. Tracking infected persons and their contacts via digital applications was discussed as a possible means to help the easing of measures, although practical measures were rejected at this time. On 1 April, a temporary Scientific Advisory Council to the Corona Behavioural Unit was set up at RIVM, composed of independent professors from the behavioural and social sciences. On 22 April, the Dutch parliament called for more diverse expertise in decision-making and in the provision of advice on COVID-19, proposing an Impact Management Team that would provide advice on transition strategies and where the social and economic effects of the crisis measures would be highlighted. On 4 May, the Outbreak Management Team introduced three pillars to anchor future advice: keeping healthcare capacity at a manageable level, protecting vulnerable people in society, and having an overview and information about the transmission of the virus. Shortly afterward, the Prime Minister announced a maximum control strategy, which could be eased if and when citizens followed the prescribed rules. Simultaneously, the Netherlands Scientific Council for Government Policy (WRR), the standing independent advisory body for government policy that had been set up to advise the Dutch government and Parliament on strategic issues with significant political and societal impacts, developed a keen focus on COVID-19. What followed was a series of initiatives aimed at providing support for the government and for parliament as they tackled the consequences of the coronavirus outbreak in the Netherlands. The emphasis was on analysis and advice for the longer term, designed to guide choices and give a direction under conditions of uncertainty, rather than to provide suggestions for ready-made policy measures. During this period, however, it was advice from the OMT that had a direct impact on policymaking, where policymakers and politicians deferred to medical expertise while the RIVM provided quantified figures to support policymaking (e.g. in terms of *R*-values, rates of infections, death rates and so on). As Hodges et al. (2022) argue, at this point in the crisis there was little opportunity or appetite for divergent or minority viewpoints, with the singular voice of the OMT (and in particular to its Chair, and Director of the RIVM Centre for Infectious Disease Control (RIVM–CIB), Jaap van Dissel) proving dominant (Figs. 1–3).

Thus, from the onset of the pandemic, the Dutch government relied on existing structures for policy advice as they had formed out of the combined *corporatist and neoliberal principles as described by* Halffman and Hoppe (2005). The government ignored the call from parliament for more inclusive and deliberative science advice. Still, the first wave in the Netherlands is characterised by a relatively high consensus on the definition of the problem and on how it should be solved, the main focus being how to stop the spread of the virus and save lives. In an interview, Jaap van Dissel, embracing his own version of the scientization of policymaking (see Christensen and Lægreid, 2022), described the management of the pandemic as a ‘relatively straight-forward and technical question’ (Keulemans, 2020). Even though this approach may have enjoyed ‘relatively high consensus’, this technocratic and somewhat unreflexive approach to policymaking was correctly criticised by the Dutch Safety Board (2022) in its thorough and careful analysis of how the Dutch Government responded to the pandemic, including its (over-)emphasis on the advice of the OMT and its prioritisation on medical advice (and on hospitals) focusing on infectious disease control, at the expense of an ongoing assessment of the wider social and economic effects of its measures, on nursing homes, but also on education, the cultural sector and small and medium-sized enterprises.

### The Speculation Phase: June 2020–August 2020: time of indulgence and public reasoning

On 1 July, the Netherlands shifted to a relaxation of measures following modelling data that had predicted a decrease in the incidence of cases and patients in need of intensive care (IC) units. Crisis control became downscaled to the regional level. The basic rules of following hygiene measures, keeping distance, staying at home, and getting tested for symptoms remained officially in place. Group sizes however were not limited anymore so long as minimum social distancing was kept. For travelling in private vehicles, the Outbreak Management Team advised the public to keep group sizes at a traceable limit. Public transport continued to require the use of a face mask as well as the 1.50-m social distance requirement. On the issue of masks, while the World Health Organisation (WHO) had advocated the use of masks as part of a comprehensive strategy of measures to suppress transmission and save lives, the OMT was more equivocal, claiming that no sound scientific evidence existed for the usefulness of masks to prevent the spreading of the virus, and continuing to refrain from advice that promoted or mandated the use of face masks in public spaces aside from on public transport. Given the participatory nature of Dutch citizens, the OMT faced criticism early on about their reluctant approach to making facemasks mandatory on a wide scale. Experts in the field such as Wim Schellekens (Visser, 2020), former chief inspector at the Health and Youth Care Inspectorate, and health economist Xander Koolman formed a public discourse via the Twitter website on advice published by the OMT which by their own words was “always constructive-critical and never dismissive” (Bremmer, 2020).

On 21 July, the Minister of Health, Welfare and Sport, Hugo de Jonge, called for an evaluation of ‘lessons learned’ by experts from previous policy advice, the goal being to extract effective actions from the first wave for optimal preparation for an expected second wave, and to learn from the experience of neighbouring countries. On this occasion, Koolman and Schellekens, as well as others included in the online discourse, met in person, having been invited by Jonge. Schellekens later stated that the outcome of this meeting was fundamentally missing its target. At the end of July, Schellekens, together with other experts who had urged Jonge to take more drastic measures, founded the Red Team—an unofficial science advisory board of independent scientists which we describe and analyse in the next section—originally composed of five permanent members. From July 2020 onwards, the Red Team started publishing their own evaluations on infection rates and proposed measures, which gained increasing recognition and visibility especially when they conflicted with the advice given by the OMT. On 31 August, the government published 117 position papers by experts aimed at providing lessons across 9 key themes, including testing and tracing, the effects of the lockdown and communication with the public (Rijksoverheid, 2020).

Speculation took place as to the likelihood of a second wave in Autumn 2020, with the Red Team urging the government to take preventative action. However, rather than proactively developing measures to prevent such a scenario, the Dutch government prevaricated. The relaxation of measures allowed space for public debate and deliberation on the measures and their effects as the initial urgency of the pandemic receded. The possibility of dealing with a second wave brought with it the threat of how to limit public life once again. The public and political debate centred on the use of face masks, the maximum size of gatherings and on the shortening of opening hours of shops. The focus of discussion moved beyond the previous priority of protecting citizens almost at all costs—with science afforded the role of providing knowledge on infection rates and the efficacy of control measures—to a wider public discussion of what measures are acceptable for society and of the trade-offs between life-saving measures with civil liberties and personal enjoyment. We can thus witness a subtle shift in the unfolding of response to the pandemic: away from a monolithic view of policy determined by the logics of (medical) science reflected in the views of the OMT and towards a more plural view of policy options each characterised by their own values and political interests, and taking into account a broader set of social and administrative considerations.

### The Second Wave: August 2020–November 2020: deliberative patterns of growing public expertise

In the Autumn of 2020, as the second wave of the pandemic came into effect, the Dutch crisis control measures necessitated an upscaling back from regional to national-level management. Stricter measures needed to be put back in place with cases rising. The OMT recognised the need to enforce rules, especially amongst hard-to-reach social groups such as students and the younger generations. In September, the upper limit within restaurants and other public social settings was set as three persons per group; employees were encouraged to work from home (unless there was no other option); a night-time curfew was recommended, and masks were advised to be worn in places such as busy shops where the 1.5 m social distancing rule is not feasible. In mid-October, the government evaluated that the measures instigated in September had not had the same impact as those delivered during the first wave. The country had reverted to too much mobility, and citizens had not adjusted their behaviour accordingly. In response, the government decided to close restaurants and pubs and to permit only take-away and online delivery. Only individual sports were allowed indoors and group sports of a maximum of 4 people outdoors. The topic of facemasks remained a polarising one, with the OMT arguing that policymakers needed to communicate a clear message about their usage. On 19 October, the COVID-19 Roadmap was published. In it, the regional approach was described as not having been successful in sufficiently decreasing the spread of the virus, leading to a greater focus on national-level approaches. Secondly, the OMT urged the importance of widely available and quick testing facilities. On 28 October, the first regulation for mandatory facemasks was drafted legislating for their use in indoor public areas, education institutions and contact-based professions.

The second wave then reached its first peak. The previous measures had demonstrated an effect, however not to the extent of similarly stringent measures that had been introduced back in March 2020. Deliberations about Christmas brought new pressures for a relaxation of measures. Communication about additional measures sought to be clear about the need and urgency of measures, while offering perspective and vision on long-term goals and strategy. The government prepped the public that COVID-19 is more of a ‘marathon than a sprint’. Up until December 2020, the Dutch government had been actively arguing against applying a total lockdown of the country, as such an authoritarian response would ‘not be Dutch’, as stated by Ferd Grapperhaus, the Minister of Justice and Security (NOS, 2020). The problem then was how to legislate and communicate the restrictive measures warranted by the second wave. Deputy Prime Minister and Minister of Health, Welfare and Sport De Jonge argued that the Dutch public needed to re-find the social solidarity that had characterised the communal response to the first wave of the pandemic in March (Van der Aa, 2020). As a partial response, subsequent press conferences further emphasised the reasoning behind the measures, the need for shared responsibility and a sense of empathy for those impacted by the measures. As Pattyn et al. (2021) attribute, the Dutch government was effective in taking on an ‘orchestrator’ role, skillfully bridging competing frames and anticipating their political effects in the art of public-meaning-making (see also Jong, 2017).

Jaap van Dissel, chair of the Outbreak Management Team, argued that the sense of urgency had changed between the first and second waves (Keulemans, 2020). Measures that had been implemented in the second wave were characterised by greater value disagreement and had less effect than in the first wave which had been characterised by greater value consensus (e.g. the values of saving lives, protecting vulnerable groups, and minimising disruption to the economy). Van Dissel argued that this change in behaviour was due to people weighing the measures differently. He stated that responsibility for deciding what measures to implement should reside with politicians, not the OMT, which should remain an advisory board. Based on the (political) evaluation of different forms of advice from a range of bodies, it was the role and responsibility of the Cabinet to determine which path to follow. These statements showed that van Dissel sought to maintain a position close to the Pure Scientist and Science arbiter positions as defined by Pielke (2007; see also section “Situating Dutch science advisory bodies in the Honest Broker framework”). The controversy around the advice of the OMT revealed the problematic nature of these positions, pushing the OMT into the role of an Issue Advocate. This also changed the relationship between the OMT and the government. Whereas at the beginning of the pandemic, with information scarce, uncertainty high, and the situation demanding urgent action, the Dutch Cabinet had accepted almost all recommendations of the OMT with minimal intervention, during the second wave with more information available, the Cabinet made its decisions based on advice from a range of sources, and sometimes even against the advice of the OMT. This disrupted the somewhat ‘cosy’ relationship that had existed between these actors creating distance (Keulemans and Hendrickx, 2020).

As governments need to represent a wide range of interests, it is not surprising that the evolution of responses to the pandemic resulted increasingly in value conflicts. Any political decision needs to be weighed against which party or interest group benefits from it and whether this is in line with the nation’s values. The Dutch characteristics of participatory decision-making and expertise, conflicting with the Dutch corporatist tradition and evidence-based policy are crucial factors in analysing Dutch decision-making processes during the COVID-19 pandemic. In effect, the polder method of ‘behind the scenes’ negotiation between designated parties had not led to consensus and authoritative governance, instead generating both plurality and public polarisation. In the following section, we analyse these tensions in more detail using the conflict between formal and informal science advice as a case study.

## Formal and shadow science advice

From the outset of the pandemic, Prime Minister Rutte made it clear that the Cabinet’s approach to controlling COVID-19 would be guided by the experts (Parool, 2020), meaning specifically the formal and solicited advice of the OMT and the RIVM. However, the provision of informal and unsolicited expert advice from the Red Team provided a ‘second opinion’ from other experts and influenced the Cabinet’s approach of making use of internal experts only. The concept of a red team has different meanings in different contexts. According to Rouse (2017), it refers to: ‘the practice of rigorously challenging plans, policies, systems and assumptions by adopting an adversarial approach. A red team may be a contracted external party or an internal group that uses strategies to encourage an outsider perspective’. The goal of a red team is to identify mistakes resulting from group thinking and confirmation bias. In corporate life, red teams are set up as a form of constructive opposition that helps keep the organisation sharp (Bremmer, 2020). Red teams have been used, for example, by US agencies such as the CIA in cyber security and geopolitical contexts, especially to identify shortcomings following the terrorist attacks of September 11, 2001.

The Red Team was formed in the Netherlands, initially on Twitter, by the Chief Inspector at the Health Care Inspectorate Wim Schellekens together with health economist Xander Koolman and field epidemiologists Arnold Bosman and Amrish Baidjoe. They were brought together by the ‘expert trajectory lessons learned’ initiative set up by Minister de Jonge (VWS). The key question was how to prevent a second wave? The four experts initially argued that they perceived at the time an urgent threat to public health (Bremmer, 2020). Around the end of July 2020, they sent Minister de Jonge a letter advocating the implementation of new and stricter measures. The letter received a lot of media attention; it was the first publicly formulated criticism by experts with an established status as policy advisors. The Red Team grew by adding eight new experts, further supported by a community with over 200 participants, covering a variety of experience and expertise from medical specialists to data experts and ex-COVID-19 patients. The Red Team was framed in the media as a shadow OMT. Wim Schellekens, the initiator of the Red Team, criticised the current composition of the OMT as lacking sufficient breadth of expertise in areas such as behavioural sciences, data analysis, primary care, and economics, only receiving advice on behavioural science from the behavioural unit of the RIVM. Nevertheless, Minister de Jonge declined their request for the Red Team to be constituted as an official advisory board. Nevertheless, the recommendations by the Red Team were taken seriously both in the media and in formal decision-making processes, in part due to the Red Team being invited to the second chamber of parliament for informal hearings (Bremmer, 2020).

Prime Minister Rutte has stated that the Red Team is ‘a club of people keeping us sharp’. The OMT, however, perceived the Red Team as a competitor. Andreas Voss, the member of OMT, feared that public compliance for COVID-19 policy would decrease due to the extra input of the Red Team, arguing that the Netherlands needed one clear direction and approach. Another OMT member, Marion Koopmans, argued that she feared the Red Team would become fuel for civil unrest (Bremmer, 2020). Another factor for discontent was that the Red Team did not consist of prestigious professors with international experience (whereas the OMT did), thus ranking lower in the academic hierarchy. The Red Team itself concurs that the OMT plays a vital role in the Netherlands. Without the OMT, the Red Team stated, “the Netherlands is lost” (Heerde, 2020). The Red Team in no way stated an interest in replacing the OMT, rather viewing their role in science advice as giving constructive side notes from different perspectives, arguing that what they would like to see is their advice at least being discussed by the OMT (Heerde, 2020).

Both the Outbreak Management Team and the Red Team expressed a worry that the other group may not always be fully objective in the provision of advice, perhaps inadvertently pursuing ulterior (political) motives. The OMT was set up as a body with formal and designated responsibilities to advise Prime Minister Rutte on the basis of sound scientific evidence at a particular point in time. In many cases, in effect, this has meant they were unable to advocate for severe measures that would restrict public life, simply because not enough solid evidence was available. The OMT developed a cultural response in which they would refuse to recommend restrictive measures in a proactive manner which could later potentially be proven to be useless given the state of uncertainties in the science. This evidence-based policymaking is a good strategy when it is clear which measures are effective and which are not. It also demonstrates a no-nonsense approach that can function as reassurance to the public when political decisions remain straightforward.

The emergence of the Red Team however made this approach less justifiable to the public. Even though the Red Team stated they were not trying to act antagonistically to the OMT, their portrayal in the media suggested otherwise: ‘Shadow-OMT’ and ‘alternative OMT’ was how the Red Team was labelled in news articles (Bremmer, 2020). The Red Team used their appearances in the media to lobby for measures based on the ‘precautionary principle (Red Team, 2020). It had tried to engage in a dialogue with the government, writing an open letter asking to diversify sources of scientific advice, which got declined. With its beginnings on the social media platform Twitter, the Red Team presented itself as more transparent than the OMT—whose meetings remained confidential. The dissenting views provided by the Red Team were publicly accessible and threatened broad regulatory compliance with guidelines that had been put in place based on OMT advice. The public now had two voices presented in the media—the Red Team and the OMT—plus the government with official guidelines typically lying in between, all complicating the provision of clear guidance to the public. The two approaches of the OMT (act only if based on sound evidence) and the Red Team (act now or regret later) lie at the heart of the Dutch inconclusiveness of guidelines and the rapid switching between degrees of the strictness of measures. While the Red Team commanded considerable authority during the period considered in this paper (January–December 2020), this diminished considerably from January 2021, following the Red Team’s advice that old and vulnerable people should be segregated from young and healthy people, to protect the former and give space for living for the latter; an initiative that became criticised in the media and which led to a clearly diminished profile (Hodges et al., 2022).

### Situating Dutch science advisory bodies in the Honest Broker framework

Roger Pielke (2007) describes five distinct ideal-type roles that science can play in policy and politics. The *Pure Scientist* is committed to research as seeking truth independently from considerations of application or practical use. Like the Pure Scientist, the *Science Arbiter* presents him or herself as removed from the policy or political process, but stands ready to assess available scientific evidence to answer particular factual questions for decision-makers. The Science Arbiter responds to requests to help narrow down choices while taking into account criteria that decision makers determine, including the values of decision-makers. The *Issue Advocate* focuses on the implications of research for a particular political agenda, aligning him or herself with these values and presenting research to advance these (value-based) interests. The Issue Advocate narrows down choices based on their own values. In contrast to the Issue Advocate, the *Honest Broker of Policy Alternatives* engages in policymaking by clarifying, and at times, expanding the scope of choice available to decision-makers in the form of alternative courses of action. Finally, there is the role of the *Stealth Issue Advocate* who disguises his or her role as ‘focusing purely on science’ while scientific facts are used as justification for a particular position rather than as clarification of the issue at hand. The Stealth Issue Advocate claims not to reduce the scope of choice while doing exactly that through the strategic framing of scientific results.

Interpreting Dutch science advice through the lens of the Honest Broker Framework may lead to the expectation that the expert orientation of the corporatist tradition strengthens the position of Science Arbiters while the consensual orientation of the participatory tradition encourages the emergence of Honest Brokers who can navigate plurality in crisis response. While this is true to some degree, we found that tensions between the neo-corporatist and participatory traditions actually led to ambiguous roles of various science actors rather than a consistent style of governance (Jasanoff, 2005) during the COVID-19 pandemic.

For example, one might place the OMT as performing a Science Arbiter role at the inception of the pandemic while also clearly identifying characteristics of the Issue Advocate as the pandemic unfolded and particularly during the second wave. The OMT was set up to provide independent scientific advice in line with the role of the Science Arbiter. At the start of the pandemic, the OMT was the sole authoritative advisory body for policymakers, providing evidence-based scientific advice in a linear manner. As the pandemic evolved, the credibility of the OMT was challenged by dissenting voices in the media and more critically with the formation of the Red Team. The discussions about the science advice of the OMT intensified without these discussions being facilitated by existing or new consultative bodies—as what commonly happens in the Dutch polder model. Attempts by journalists to get clarification on how the OMT dealt with the plurality of scientific advice and/or criticism on their functioning were typically deflected by formalistic statements about their task as being that of weighing all the evidence (taking the Science Arbiter stance) or stressing that they only worked with the knowledge brought in by OMT members (suggesting other disciplines were of lesser relevance, indicative of a Pure Scientist role).

However, the OMT did not continue entirely on the same footing as at the beginning of the outbreak, communicating rather in ways that are closer to an Honest Broker role by acknowledging the various interests that exist in society but without giving up its role as a Science Arbiter. OMT members emphatically expressed that their scientific expertise was the prime source of policy advice. This narrow understanding of pure scientific ‘objectivity’ that the OMT is ascribing to itself is a fallacy in so far as it omits its own value-based propositions (Slob and Staman, 2012): in this case, the assumption that the best way to control the pandemic was through evidence-based infectious disease control measures, that such measures needed to be supported by evidence typically in the form of quantitative measures (e.g. *R*-number, infection rates, ICU and hospital admissions), that compliance would be assured through the promise of future relaxation, and that the positive consequences of the carrying through the measures would outweigh the (soft and hard to quantify) negative impacts of the measures, including the psychological well-being of vulnerable people and socio-economic impacts (Dutch Safety Board, 2022). However, decision-making in a crisis is influenced by a diversity of perspectives and values, including those of medical science. The Science Arbiter role is arguably most useful for decision-makers when values are aligned and uncertainty is low, both conditions that do not apply to the COVID-19 crisis. By applying purely scientific medical advice, and failing to recognise the legitimacy of alternatives, the OMT approach is destined to result in conflict as it does not authorise the legitimacy of alternative values and perspectives regarding both the social treatment of the uncertainties underpinning the science and the efficacy and impacts of measures. Equally important, such a perspective and its focus on short-term crisis communication pay little attention to societal structures and the complexity of public concerns.

The Red Team was set up in opposition to the OMT. Similar to the OMT the values underpinning the Red Team were to open up choices aimed at saving lives. By doing so Red Team members positioned themselves in the same Science Arbiter role as the OMT. The Red Team, however, was also an Issue Advocate, as it embraced this profile by arguing for wider protection of vulnerable groups in contrast to the then-perceived hegemonic focus on individual freedoms and liberties which the government had followed with its policy of the intelligent lockdown. Although Red Team members were taking value-laden positions more explicitly than OMT members, commonly shaped by the value of precaution, their perceived function was ostensibly to put pressure on the government to facilitate a more deliberative model for science advice in which Red Team members, similar to OMT members, could operate in ways that would either fit with the Science Arbiter role or the Honest Broker role. Indeed, it was this attempt to pluralise scientific advice that was actively resisted by the OMT as failing to comply with the ‘rules of the game’ (Bogaards, 2020): i.e. that scientific advice should be solicited by the government, given in secret and with a singular voice.

### Case study: The contestation of scientific advice on facemasks

To illustrate the tensions between the Outbreak Management Team and the Red Team, we analyse the contested topic of whether or not to make facemasks mandatory in Dutch public spaces. In May 2020, the OMT concluded in one of its first advisory statements that there was insufficient evidence to support the effectiveness of non-medical face masks in public spaces. In their advice, the OMT stressed that while facemasks may provide protection where minimum distance could not be maintained, rigorous social distancing was ranked as the more effective strategy, fearing that people would not adhere to social distancing if obligated to wear masks. The OMT labelled this risk as ‘illusive security’. In June 2020, facemasks became mandatory in all Dutch public transport facilities given that minimum social distancing was not always an option. The OMT constructed their opinion on the basis of advice from the WHO, urging for the use of facemasks in settings where community transmission is likely. The WHO, however, also stated that there was as yet no sound scientific evidence on the efficacy of face masks, which the OMT took as indirect evidence for their hypothesis of facemasks as plausibly ineffective.

In July 2020, the Outbreak Management Team stated that most COVID-19 virus transmissions took place in private settings, deeming facemasks in public spaces once more to be redundant. From July onwards, the Red Team criticised the lack of action taken to prevent the spread of the virus in public areas. Facemasks should be made mandatory for any jobs that rely on social contacts, the Red Team argued, such as hairdressers and grocery stores. Even more, the government should start a campaign educating civilians about the proper use of masks. In August 2020, the Red Team contradicted the advice of the OMT and their statements on the (plausible) ineffectiveness of facemasks while at the same time making them mandatory on public transport. For the Red Team, this discrepancy would likely cause confusion and public distrust. The Red Team advocated, following the WHO and ECDC (European Centre for Disease Prevention) advice, to make masks mandatory in *all* public spaces. This point was reiterated in subsequent open letters in August 2020 regarding the re-opening of schools where the Red Team argued for the wearing of masks for children aged 6 and above and for teachers.

In September 2020, the Red Team highlighted new scientific findings on the aerosol transmission which claimed that facemasks hold back the transmission of the virus, at least partially. Contrary to the OTM, they argued that masks do not provide ‘illusive security’ but a reminder and nudge for citizens to follow measures in public areas. Important here is that the Red Team justified their position through the use of representative opinion poll data, where 72% of the surveyed public expressed a preference for face masks to be made mandatory in public places, opening up the discussion not only to what the science says but also what the public wants. In October 2020, the OMT demanded clarity from policymakers on the (mandatory) use of facemasks, on how this was to be communicated to the public as well as correct usage. At the same time, the Red Team released an analytical report based on a collection of scientific studies on the role of aerosols and drop infections in spreading the virus as a basis to support their position for as many protective measures as possible to stop the spread of the virus.

The conflict between the OMT and the Red Team belies different social treatments of uncertainty. The OMT relied on a model of sound science, stating that there was insufficient scientific evidence to justify the wearing of masks. From the start, the OMT seemed divided explicitly stating in their advice that certain issues had not reached a consensus among members. The OMT argued from a rational and logical point of view; it assumed that there were no conflicting values and that science alone could adjudicate the effectiveness of using a face mask. However, conflicts in values did exist, and thus unsurprisingly, science was not able to provide a clear answer, leading to several studies coming to contradicting conclusions. As a result, the scientific debate turned into a political debate and the issue of face masks became increasingly discussed in the media and in society as a political issue (Keulemans, 2020).

From September 2020, the Red Team lobbied for the mandatory use of facemasks to prevent a second lockdown. It took until November 2020 for the government to catch on and make steps toward mandatory usage. From December 2020 it became mandatory to wear a face mask in a public spaces. The length of the discussion is evidence that it had long moved from simple linear science arguments into more complex and value-driven discussions. Both the OMT and the Red Team substantiated their demands for or against a face mask rule with respective scientific studies. As a result, the institution of science had lost in part its perceived objective status. This analysis characterises the approaches of the two organisations: one as proactive (Red Team), the other reactive (OMT), both able to argue that their decisions had been made based on scientific evidence and to arrive at contradictory advice. To conclude this case, while the Dutch Government relied heavily on their own institutionalised scientific advisors when considering the implantation of face masks (the OMT), the external science advice coming in from the Red Team can be seen as having a nudge on the decision-making process in the autumn of 2020. Nevertheless, the ‘fall’ of the Red Team arising from its advice of separating old from young people in January 2021, can be seen as a case of the ‘proactive stance’ going wrong, or at least leading to very controversial conclusions.

## Conclusion

The Dutch response to the COVID-19 pandemic exhibits a set of unique national characteristics but also reflects wider dynamics of contestation that are highlighted by other case studies of this special issue (such as the role of formal and informal advisory structures). The Dutch playbook and its implementation during the pandemic have been shaped by science advisory structures that emerged from a corporatist model, refurbished in the 1990s on neoliberal principles. As explained in the section “Introduction”, one of the effects of the refurbishments was a proliferation of ‘knowledge centres’ around specific societal issues that provides opportunities, at least in principle, for deliberation with a variety of stakeholders (Halffman and Hoppe, 2005). The COVID-19 pandemic showed the limitations of this structure. The neoliberal principles strongly reduced a central coordinating mechanism for the decentralised health centres, including elderly homes and hospitals. There was no clear command structure between the ministry and the 25 Safety Regions, that in turn each developed different policies, implementation strategies and work routines. Or, in the words of the Safety Board (2022, p. 85), ‘the boards of the Safety Regions are used to being politically legitimised to make their own local choices.’ However, this did not imply that coordination between Safety Regions was absent, on the contrary. The Safety Board observed in the same report that the regions between them are well-networked and frequently have common training and exercises for crisis situations.

This framing provides interpretative tools for understanding the Dutch response to the COVID-19 pandemic in line with this tradition, with one major exception. Whereas the Dutch government under ‘normal’ conditions would facilitate the deliberative polder model to allow various interests groups and experts to find a common ground in dealing with complex issues, science advice for dealing with the COVID-19 pandemic was exclusively solicited from the official Outbreak Management Team, in line with the playbook for such calamities. On the one hand, the playbook and its implementation demonstrate the institutionalisation of an expert-driven response that strives to be evidence-based and that is embedded in a complex network of science advisory bodies. On the other hand, the early phase of the pandemic was accompanied by emphasis on personal responsibility and individual choices rather than coercive measures. This constellation was successful in the early stage of the pandemic as participatory elements did not substantially challenge the corporatist consensus. The second wave of the COVID-19 pandemic exposed the fragility of this constellation. Although urgency levels again increased under the pressure of quickly rising infections, much more was known about the virus, its transmission pathways and disease symptoms, as well as about the effects, trade-offs and externalities of control measures. Moreover, the quickly amassing knowledge was distributed across various scientific disciplines and professional bodies. As the pandemic evolved, solidifying corporatist consensus appeared increasingly problematic as expert opinions diverged and were met with general public discontent about the state of pandemic governance. In other words, the centralised response of the Dutch government reversed the decentralisation it had advocated for and implemented over the years. Not only the corporatist consensus but also the policy focus on individual decision-making and personal responsibility became destabilised in this process. In the wake of the second wave of the COVID-19 pandemic, the Netherlands implemented a mandatory curfew, mask mandate, and lockdown that were largely comparable to those of other European countries.

By interpreting this destabilisation through the combination of the frameworks provided by Halffman and Hoppe (2005) and Pielke (2007), we have provided a wider analysis of the institutional structures and dynamics of Dutch science advice. Recognising different styles of governance (Jasanoff, 2005), the Netherlands may have been expected to generate a more successful and less contested crisis response than highly polarised environments such as the United States and the United Kingdom. Commonly characterised through a unique “polder model”, Dutch governance may have been expected to navigate the COVID-19 pandemic both through high regard for expertise in the corporatist tradition that could ensure the success of Science Arbiters and through the strong focus on consensual decision-making in the participatory tradition that could facilitate the emergence of Honest Brokers. Nevertheless, while at the macro level, the Netherlands indeed has a distinct style of governance that can in principle facilitate honest brokers and deliberation, at the meso level we found that the Dutch playbook for responding to pandemics reflected more the corporatist tradition with little designated space for deliberation of policy options outside a restrictive model of infectious disease control. Perhaps then, unsurprisingly, at the micro level of crisis management, we found that the deliberative style of governance quintessentially favoured by the Dutch as a mark of identity and tradition largely broke down under the pressure of the pandemic, first through the dominance of corporatist responses, and subsequently through pluralised polarisation. In this sense, the Dutch model seems to articulate a largely unfulfilled promise of better pandemic governance.

Such expectations of rational expert-oriented and consensual governance were not fulfilled. As expert advice did not succeed in controlling the pandemic, actors and their roles became increasingly contested. Formulated through the lens of the Honest Broker framework, core bodies did not function as clear Science Arbiters or Honest Brokers but pushed established institutions such as Outbreak Management Team towards issue advocacy and facilitated the emergence of novel proactive actors such as the Red Team. While the Dutch style of expert-oriented and consensual governance may have been seen as a promising model for crisis response, it should give pause for thought that even the Dutch were not able to implement it successfully. Some failures may reflect that contemporary Dutch society is actually not well-represented through the cliché of harmonious plurality in the Dutch polder and that this cliché obscures how societal polarisation in the Netherlands follows similar patterns as other European countries. However, the Dutch context also highlights how difficult it can be to implement successful Science Arbiters or Honest Brokers during crises that are characterised by rampant uncertainty and in which expert advice does not directly translate into salient success. Compared to countries such as the United States and the United Kingdom, the Netherlands has more established traditions of maintaining trust in expertise while fostering participatory processes of establishing consensus. The force of the COVID-19 pandemic, however, tested the limits of these traditions and very rapidly generated a contested terrain of Issue Advocacy rather than deliberation facilitated by Honest Brokers.

Finally, it is important to note that our analysis clearly lies in tension with the currently dominant public discourse about the position of scientific advice in COVID-19 policymaking. While the dominant view, recently articulated by the Dutch Safety Board (2022) and endorsed by Parliament, calls for a clearer separation of powers between institutional structures and cultures of scientific advice and public policymaking, our analysis makes clear that this would turn back the clock in problematic ways. Although no one would disagree that an epidemic outbreak involving a new type of pathogen requires prioritisation of highly specialised expertise, the co-emergence of a range of other issues as well as learning effects of crisis institutions require not only broadening of expertise but also additional efforts to weigh and reflect on expert knowledge. The Netherlands has a tradition of involving policymakers and other societal actors in such a process. Secondly, the strong focus on government policy and science advice at the national level discards the decentralisation of such processes in recent decades. Our analysis is that such separation and re-centralisation will do little to improve legitimacy and lead to authoritative governance. Rather, what is required according to our analysis, is the need for greater reflexivity, value recognition and inclusive participation as guiding features to structure scientific advice.

## Supplementary information

Supplementary Figure S1

## Footnotes

1 We are grateful to one of the reviewers of this article for bringing this point to our attention.
