# Supplementary material for: The Dutch see Red: (in)formal science advisory bodies during the COVID-19 pandemic
Source: Humanit Soc Sci Commun. 2022 Dec 24;9(1):464. doi: 10.1057/s41599-022-01478-w (PMC9789726; doi:10.1057/s41599-022-01478-w)
Supplement: Supplementary file 1 — Supplementary Figure S1 [file 41599_2022_1478_MOESM1_ESM.pdf]

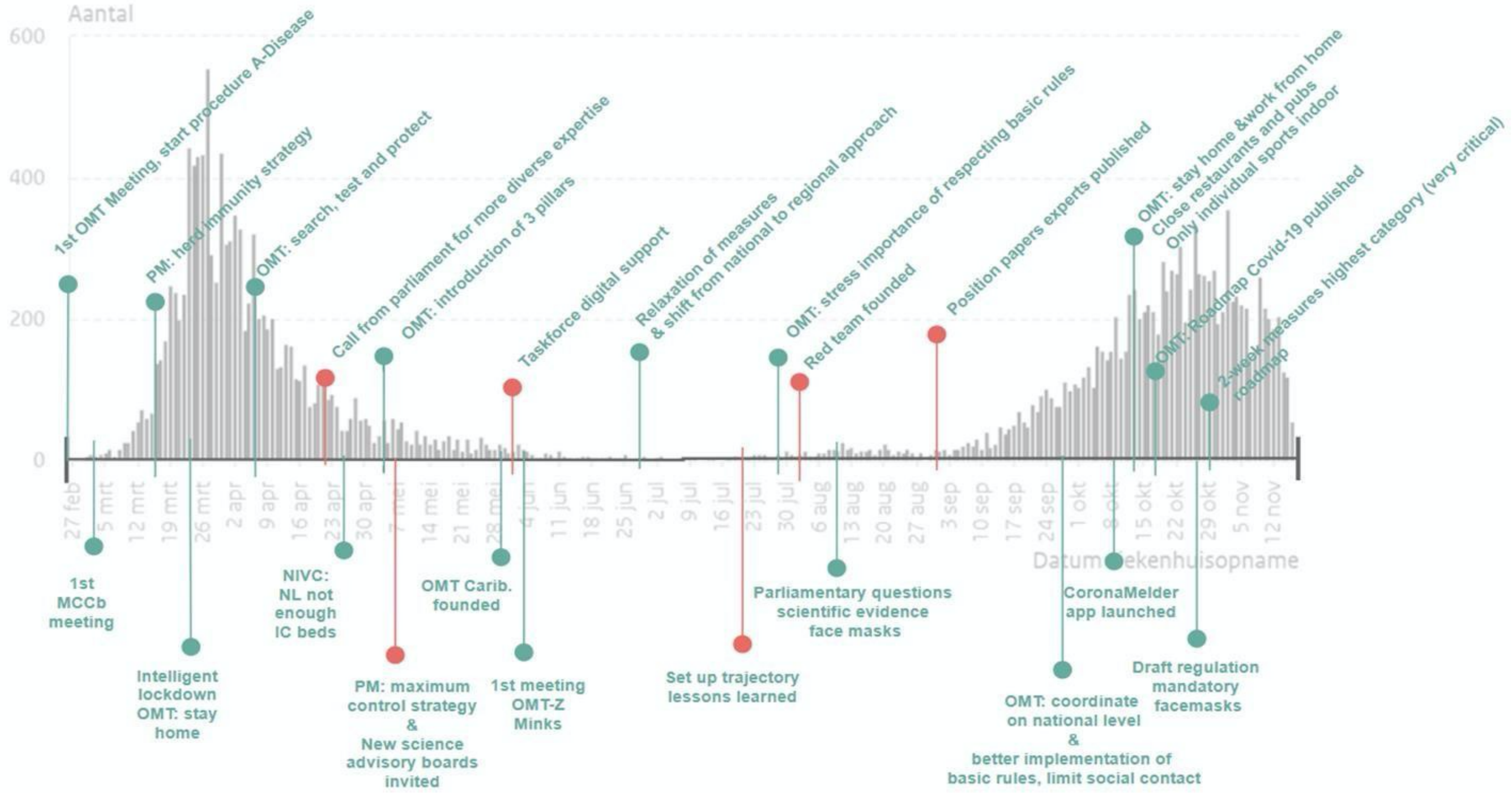

Source: NICE, 2020

Supplementary Data D1. Timeline Covid-19: March 2020 – December 2020

24st January 2020 - 1st OMT meeting and start procedure A-disease

- The first OMT (56) meeting is held on the 24th of January 2020. The meeting entails a briefing on the facts that are available, with a description of the background and the situation in Wuhan. The risk for the Netherlands is classified as moderate. The risk of spreading is not regarded as high since all travel out of and to China has been stopped. On the 29th of January 2020, the procedure for Adisease outbreak is initiated.

3rd March 2020 - 1st MCCb meeting

- The Ministerial Commission Crisis Control (MCCb), is under the presidency of the prime minister, decides about the measures and facilities as a whole focusing on a coherent approach to prevent further spread of the coronavirus and its impact on society.

16th March 2020 – Prime Minister: herd immunity strategy

- In a speech of prime minister Mark Rutte, a couple of options to deal with the novel Covid-19 virus are addressed. He argues that the options are herd immunity, total lockdown or no intervention at all. He advocates a herd immunity strategy.

23rd March 2020 - Intelligent lockdown, OMT: stay home

- The reason not to impose a complete lockdown is explained by director CIb and the prime minister. They argue that in case of a total lockdown, the virus cannot spread easily, however, also nobody builds immunity. Thus, the young, healthy population would get infected, and the elderly and vulnerable groups would be protected. In this way, the prime minister argues, a controlled approach of building immunity can be achieved. This receives a lot of backlash from society, it is said that it would potentially cost lives which could have been saved (Holdert, 2020).

6th April 2020 - OMT: search, test and protect

- The OMT stresses the importance of searching for infected persons, testing and protecting the elderly and vulnerable groups. They identify that contact tracing is the most important factor in order to quickly identify cases and isolate these. The issue of tracking via digital apps is brought up. The easing of measures is discussed and rejected.

22nd April 2020 - Call from parliament for more diverse expertise

- On the 22nd of April 2020, a motion is adopted unanimously by parliament, requesting that the government should include a greater variety of experts regarding Covid-19 advice. They propose an Impact Management Team which could help to advise regarding transition strategies in which the social and economic effects of the crisis measures can be highlighted.

24th April 2020 - NIVC: NL not enough IC beds

- The NIVC publishes a calculation of the intensive care unit beds needed in order to let herd immunity run its course; the Netherlands falls short by roughly 400 beds.

Minister VWS responds by stating the need for increasing the hospital capacities as well as the IC units.

4th May 2020 - OMT: introduction of three pillars

- The OMT sets three pillars on which it shall consider its advice:
  - Keeping health care capacity at a manageable level – hospitals should be able to provide care to Covid-19 patients and regular patients.
  - Protect vulnerable people in society.
  - Have an overview and continue to be informed about the development of the spread of the virus.

6th May 2020 - PM: maximum control strategy and new science advisory boards invited

- The strategy of maximum control is based on two goals: protecting the elderly and vulnerable groups, and make sure IC capacity is not overburdened. The prime minister states that the Netherlands can open up again but only if everyone acts sensible and keeps following the prescribed basic rules. He states that the goal of this strategy is that herd immunity is built. But he immediately also stresses that herd immunity is not a goal in itself, rather an effect. But this line of thought continues with a couple of advisors to the government (Holdert, 2020).
- Prime minister Rutte and Minister VWS de Jonge responded positively to the motion of parliament on the 22nd of April 2020. Minister de Jonge argues that the social-economic terrain is very broad. Therefore, the cabinet is not basing their advice on one advice body, it wants to include more perspectives. The cabinet chooses not to compose an advisory board but utilises recent (existing) initiatives.
- These are independent of the cabinet and can decide themselves whom to involve in their work. They share their advice unsolicited or upon request if they deem relevant for the Cabinet or Chamber. The emphasis for this organisation is to analyse and advise on the longer term, not about direct measures. They already advised the government, but this will be intensified during the course of the Covid-19 pandemic. The following science-advisory bodies are selected: Think Tank Corona crisis (SER), Covid-19 consultation Planbureaus (Planning bureaus SCP, CPB, PBL and RIVM) and RIVM and cooperation with local decision-makers. Covid-19. The planning bureau SCP looks at the consequences of the Covid-19 pandemic for the quality of life. The CPB looks at the economic consequences on the short and long term. The PBL looks at the consequences for climate, energy, nature and agriculture. These are all national governmental research institutes.
- Even though the advice is incorporated by policymakers, it seems that the OMT advice is more dominant in policymaking. On the 1st of April, a scientific advisory board Corona behavioural unit of RIVM is created which exists out of independent professors.

30th May 2020 – Creation of an OMT-Caribbean

- An OMT Caribbean is created to advise the Dutch Caribbean (Aruba, Bonaire, Curacao, Saba, SintEustatius and Sint-Maarten). The OMT Caribbean is a special construct which falls under the responsibility of the RIVM.

2nd June 2020 – Taskforce digital support

- With the launch of the concept of the CoronaMelder app, the Taskforce Digital Support in controlling the coronavirus, Taskforce(digital) behavioural sciences and a guidance committee are established.

3rd June 2020 - 1st meeting OMT-Z Minks

- The OMT-Z is gathered to deal with zoonoses and Covid-19 due that there is a transmission between humans and minks. It sets up an Early Warning System. They advise that farms which have been infected should isolate and kill infected minks and the government should shut down infected mink farms. Farms which have not been infected should follow strict hygiene rules.

1st July Relaxation of measures & shift from national to a regional approach

- The modelling of cases gave an indication that the incidence of cases decreased as with this also the patients in need of IC units and hospital care. The basic rules remain in place:
  - Stay at home when showing symptoms and get tested
  - Follow hygiene measures
  - Keep the distance of 1,50 metre
  - Work as much as possible from home
- For any types of gatherings, the OMT makes a distinction for the maximum group size. If any participant has to register and a minimum distance can be kept inside/outside, the OMT does not limit group sizes. In case of no air circulation in the facilities, the maximum number of participants is to be kept at 100 for inside gatherings and 250 for outside gatherings.
- Children in primary and secondary schools are excluded from the 1,50-metre distance rule. Sports and cultural activities are excluded as well. Public transport continues to require a face mask as well as 1,50-metre distance. For travelling in private vehicles, the OMT advises the public to keep group sizes at a traceable limit.
- Regarding masks, the OMT states that the WHO declared the indirect usefulness of masks in preventing an infection. The OMT keeps to its statement that no sound scientific evidence exists over the usefulness of masks to prevent the spreading of the virus. It continues to refrain from stimulating the use of face masks to the public.

21st July Set up expert trajectory 'lessons learned'

- The minister de Jonge announces the implementation of an evaluation by experts of all previous policy advice given out during the pandemic. The goal is to extract all effective actions of the first wave in order to be prepared for an expected second wave. The Netherlands also aims to learn from neighbouring countries by evaluating their approaches through case studies of crisis control approaches. Experts were invited to share their opinion on the following topics: testing and tracing, intensive care units, effects on regular health care, general lockdown measures, long term care, effects of lockdowns on vulnerable populations, long-term care, intensive care units, effects on the regular health care system, public communication and Dashboard

28th July OMT: stress importance of respecting basic rules

- The OMT makes it clear that even though medical face masks may help in reducing the risk of droplet infections, non-medical masks do not. Their main point is that Dutch citizens ought to comply with the minimum distance of 1,5 metres at all times and that face masks may be used as an additional tool if the distance cannot be kept. Most infections have shown to take place in personal settings. The OMT stresses the importance of respecting the basic rules put in place of everyday social interactions. The size of groups should be kept at a minimum at which keeping the distance can be maintained at all times.

End July: The Red Team is founded

- Before this point, the Red Team was not set statically on their members and their organisational structure. By now it has settled on at least five permanent members, as elaborated above. The advice of the Red Team has gained more and more recognition over the course of the last months coupled with conflict from side of the OMT as described in the introduction of this document.

11th August: Parliamentary questions regarding scientific evidence of face masks

- The OMT states again that there is no conclusive evidence on the usefulness of face masks in social settings. It stresses again that keeping a minimum distance is the most effective way of preventing spreading of the virus. They base their position on the necessary training of correct use of face masks in order to be effective. They refer back to the basic measures regarding keeping distance, staying at home with symptoms, testing and working from home.

31st August: Position papers were published

- The aforementioned papers of trajectory 'lessons learned' by experts are published. They encompass thirteen papers of different experts stating their assessment of dealing with the pandemic.

28th September: OMT: coordinate on national level & better implementation of basic rules, limit social contact

- The pieces of advice of the OMT focuses on national guideline coordination. Stricter measures need to be put in place. The rules need enforcement among social groups such as students and younger generations. A maximum of 20 people indoors is allowed. Within restaurants, the upper limit is three persons per group. A night curfew is in discussion. Masks might be advised to be mandatory in shops and supermarkets where the minimum distance cannot be kept.

8th October CoronaMelder app launched

- National introduction of the CoronaMelder app. The app is supposed to function as a helpful tool to trace contacts and sources of infections and therefore contain the spread more effectively. Reporting as well as registration/download of the app is voluntary.

12th October OMT: stay home & work from home; closing of restaurants and pubs; only individual sports indoors

- The measures taken on the 28th of September do not have the same effect as in last June. There is too much mobility within the country, and it seems that people are not

changing their behaviour. The advice is to stay home and work from home. Citizens shall avoid hotspots and limit travelling. A maximum of 3 guests per household per day is advised. Restaurants, pubs and bars are closed, take away and delivery is allowed. Only individual sports are allowed indoor and with a maximum of 4 people outdoors. Facemasks are polarised, and the OMT argues that policymakers should communicate a clear message about the use of face masks. News on testing: OMT is positive about antigen testing.

19th October OMT: Roadmap Covid-19 published

- A roadmap is developed indicating different levels of the outbreak. The regional approach has shown to be slower in containing the spread. Therefore, the roadmap focusses more on a national level in which expectations on regional level measures are also expressed. Furthermore, OMT expresses discontent regarding the limited current parameters (google and apple mobility trends) available to them whereas other European countries have access to more phone data.
- The current testing strategy should be scaled up. There should be faster testing procedures and more testing facilities. A number of parties are working on faster testing procedures, but the OMT would like to have an overview of these.

28th October Draft regulation mandatory facemasks

- Multiple ministries have worked on a draft regulation of making facemasks mandatory in indoor public areas, education institutions and contact-based professions.

29th October 2 week measures highest category (very critical) roadmap

- The measures of 28th of September and 13th of October have been effective. However, the same effect as in March was not reached. Therefore, for a period of 2 weeks, the measures accompanying 'very critical' of the roadmap will be put in place. For festive holidays (and if it can be permitted), the government should set policy about the relaxation of measures. Society should prepare itself that Covid-19 requires long-term adaption (like a marathon). On the one hand, communication about additional measures should be clear-cut about the need and urgency, however, the notion of offering perspective and vision on long-term should also be included. Migrant workers remain a vulnerable group for Covid-19. Antigen testing is not recommended.
